# Cardiac Allograft Vasculopathy: Challenges and Advances in Invasive and Non-Invasive Diagnostic Modalities

**DOI:** 10.3390/jcdd11030095

**Published:** 2024-03-21

**Authors:** Moaz A. Kamel, Isabel G. Scalia, Amro T. Badr, Nima Baba Ali, Juan M. Farina, Milagros Pereyra, Mohammed Tiseer Abbas, Ahmed K. Mahmoud, Robert L. Scott, David E. Steidley, Julie L. Rosenthal, Lisa M. Lemond, Kristen A. Sell-Dottin, Brian W. Hardaway, Timothy Barry, Ming Yang, Chieh-Ju Chao, Clinton E. Jokerst, Chadi Ayoub, Reza Arsanjani

**Affiliations:** 1 Department of Cardiovascular Medicine, Mayo Clinic, Phoenix, AZ 85054, USA; 2 Department of Cardiothoracic Surgery, Mayo Clinic, Phoenix, AZ 85054, USA; 3 Department of Radiology, Mayo Clinic, Phoenix, AZ 85054, USA; 4 Department of Cardiovascular Medicine, Mayo Clinic, Rochester, MN 55905, USA

**Keywords:** cardiac allograft vasculopathy, angiography, echocardiography, nuclear imaging

## Abstract

Cardiac allograft vasculopathy (CAV) is a distinct form of coronary artery disease that represents a major cause of death beyond the first year after heart transplantation. The pathophysiology of CAV is still not completely elucidated; it involves progressive circumferential wall thickening of both the epicardial and intramyocardial coronary arteries. Coronary angiography is still considered the gold-standard test for the diagnosis of CAV, and intravascular ultrasound (IVUS) can detect early intimal thickening with improved sensitivity. However, these tests are invasive and are unable to visualize and evaluate coronary microcirculation. Increasing evidence for non-invasive surveillance techniques assessing both epicardial and microvascular components of CAV may help improve early detection. These include computed tomography coronary angiography (CTCA), single-photon emission computed tomography (SPECT), positron emission tomography (PET), and vasodilator stress myocardial contrast echocardiography perfusion imaging. This review summarizes the current state of diagnostic modalities and their utility and prognostic value for CAV and also evaluates emerging tools that may improve the early detection of this complex disease.

## 1. Introduction

Cardiac allograft vasculopathy (CAV) is a progressive obliterative vascular disease that impairs blood flow to transplanted hearts, leading to late graft failure [1]. Affecting approximately 30% of patients at five years post-transplant and almost 50% at 10 years, CAV is considered the leading long-term cause of cardiovascular mortality in heart transplant (HTx) patients [2,3]. The pathophysiology of CAV is complex and not yet fully understood, but it is generally considered a predominantly immunologically mediated disease, hastened by cardiovascular risk factors that may be associated with immunosuppression [4]. Immune-mediated remodeling of the vasculature in transplanted hearts leads to endothelial injury, vascular cell proliferation, and fibrosis, resulting in stenotic vessels with impaired perfusion [5]. The risk of developing CAV is highly increased with immunological risk factors such as the occurrence of acute rejection, the presence of alloantibodies, and infections like cytomegalovirus [6]. However, traditional non-immunologic cardiovascular risk factors including hypertension, diabetes mellitus, and hypercholesterolemia also play an important role [7]. HTx patients are often asymptomatic with CAV as cardiac allografts are denervated, preventing patients from experiencing classic angina [8]. Instead, recipients often have nonspecific symptoms or present with reduced left ventricular ejection fraction (LVEF) and heart failure [5,8]. Unless recognized and managed early, many of these patients can present with silent myocardial infarction, syncope due to conduction deficit, arrhythmia, or sudden death [9].

Given the subtle presentation of CAV, regular periodic surveillance is recommended beyond the first year after HTx [8]. The gold-standard diagnostic technique is invasive coronary angiography (ICA), either with or without intravascular ultrasound (IVUS), which carries a risk of contrast-related kidney injury and procedure-related vascular lesions [10]. Often, however, transplant patients with normal ICA have been found to have a high prevalence of CAV on autopsy [11]. Over the past decades, non-invasive modalities, spanning from rest echocardiography to nuclear imaging, have been investigated to improve the early detection of CAV [1,5]. The purpose of this study is to review the previously accepted gold-standard modalities and explore the currently available non-invasive options for the early detection of CAV and the follow-up of HTx patients.

## 2. Invasive Modalities

### 2.1. Invasive Coronary Angiography

**Figure 1:**
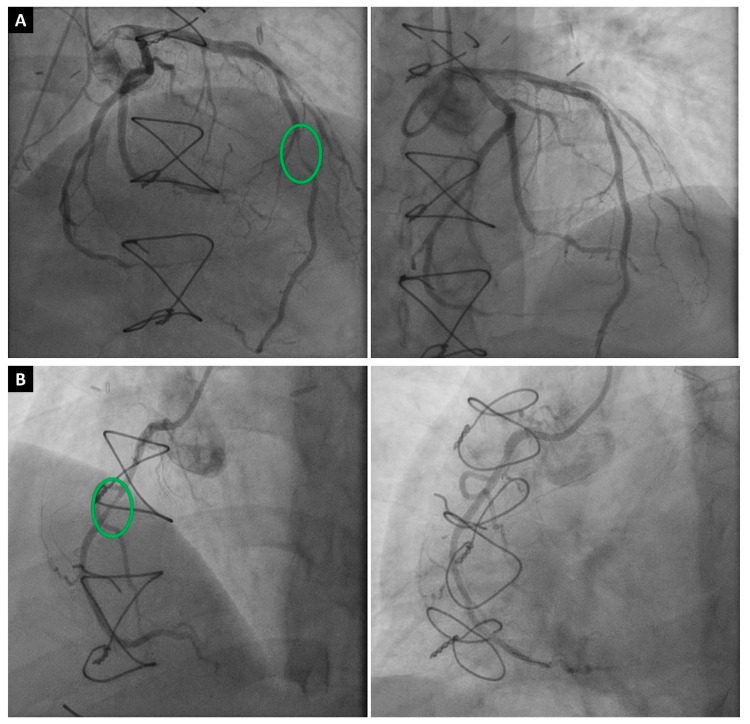
A 53-year-old male patient who developed severe CAV two years after HTx. (**A**) Successful intervention of the middle left anterior descending artery (green circle) with one drug-eluting stent (preintervention stenosis was 90%). (**B**) Successful intervention of the middle right coronary artery (green circle) with one drug-eluting stent (preintervention stenosis was 70%).

The International Society for Heart and Lung Transplantation (ISHLT) recommends ICA as the gold-standard method for the diagnosis and surveillance of CAV (Figure 1) [3]. Typically, ICA is recommended one month after HTx and then annually or biannually; however, it may be required less frequently if no apparent symptoms or signs of CAV are found three to five years after HTx [12]. Based on angiographic findings and evidence of graft dysfunction, the ISHLT criteria classify CAV into four grades, as follows: absent (CAV_0_), mild (CAV_1_), moderate (CAV_2_), and severe (CAV_3_) [2,3,13] (Table 1). Although widely available in most institutions, ICA has several considerable drawbacks. One significant limitation of ICA is its reported insensitivity to detect diffuse concentric lesions, as seen in early CAV, as it cannot visualize beyond the arterial lumen [12,14].

Therefore, ICA is commonly performed in conjunction with another intravascular imaging modality that can examine the vessel wall, such as IVUS or optical coherence tomography (OCT). These modalities have been shown to detect severe concentric intimal hyperplasia in cases where ICA images of the same vessel show normal-appearing vascular lumen [15]. Furthermore, ICA can only identify luminal stenosis but is not capable of wall and lesion characterization. The addition of IVUS and OCT help to characterize the lesion clearly [14,16]. In a study by Tuzcu et al., the sensitivity and specificity of ICA alone for detecting CAV were 43% and 95%, respectively, for patients with confirmed CAV on combined ICA with IVUS [14]. This study highlights the diagnostic value of combining ICA with IVUS for detecting early CAV.

Frequently obtained during ICA, fractional flow reserve (FFR) is a technique used to measure the pressure difference across a stenotic region in a coronary artery, which assists in determining the severity of the stenosis from a functional perspective and whether it affects the blood flow to the myocardium [17]. FFR is calculated as the ratio of the pressure after the stenosis to the pressure before the stenosis during maximum blood flow (hyperemia), usually elicited by intracoronary adenosine injection [18]. In the presence of coronary stenosis, there is already vasodilation of the coronary artery distal to the stenosis, so administration of adenosine fails to cause significant further dilation distally and, thus, the FFR is reduced [19]. In the absence of significant epicardial stenosis, a decreased FFR after adenosine administration could indicate microvascular disease [20]. Hiraishi et al. demonstrated this in a study of 33 pediatric patients with Kawasaki disease, reporting reduced FFR to be correlated with histopathologic and angiographic evidence of microvascular disease [21].

Quantitative flow ratio (QFR) is a newer technology that uses a three-dimensional geometric reconstruction of conventional angiographic and intravascular images to compute a surrogate for FFR [22]. In a retrospective study of 22 HTx patients, Shah et al. found a QFR threshold of 0.88 to be more accurate than ICA and IVUS in predicting subsequent CAV development or progression, with a sensitivity of 94% and a specificity of 67% [10]. However, more studies are needed to confirm these findings and compare QFR to other screening modalities.

### 2.2. Intravascular Ultrasound

**Figure 2:**
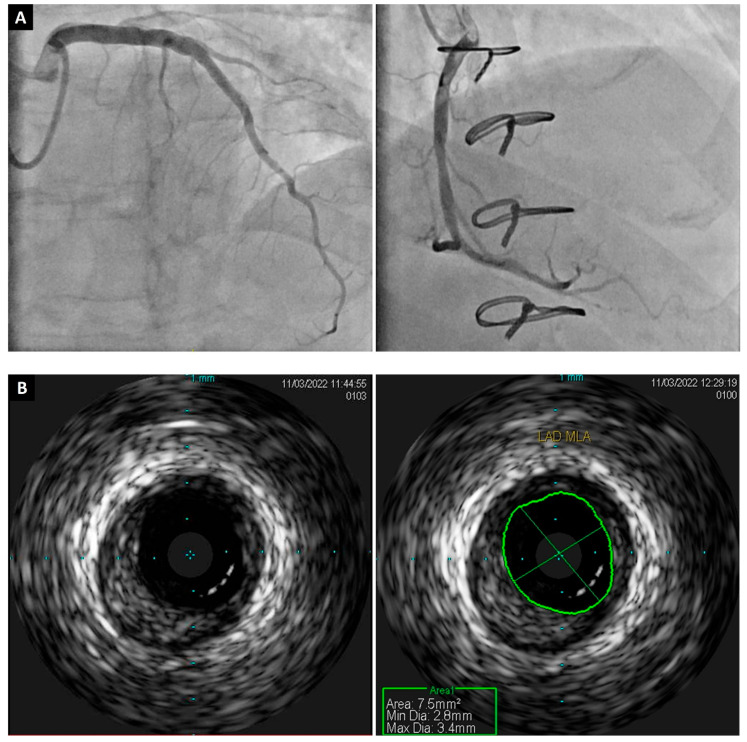
A 62-year-old male patient who developed CAV 10 years after heart transplant. (**A**) Conventional ICA showing no significant stenosis; middle left anterior descending artery is 10% obstructed by diffuse disease and middle right coronary artery is 20% obstructed by diffuse disease. (**B**) IVUS was performed in mid left anterior descending artery and confirmed the presence of significant atherosclerosis. *CAV, cardiac allograft vasculopathy; ICA, invasive coronary angiography; IVUS, intravascular ultrasound*.

Typically performed during ICA four to six weeks after HTx and again at the one-year mark, IVUS is recognized for its sensitivity in assessing the anatomy of epicardial coronary arteries, including the thickness of intimal and adventitial walls (Figure 2) [23]. The use of IVUS to screen for CAV has increased significantly over the past 20 years because it allows for the earlier detection of intimal thickening before this process may be apparent on ICA [15]. Multiple studies have demonstrated the prognostic impact of the IVUS-based parameter, maximal intimal thickness (MIT), in the early detection of CAV [24]. In these studies, an MIT greater than 0.5 mm was associated with higher rates of mortality, cardiac events, and revascularization four years after HTx [25]. Although combining IVUS with ICA is the current standard for the early detection of CAV, it has its own limitations of an increased risk of complications during angiography and an increased time and cost of the procedure, along with limited vessel coverage [24].

### 2.3. Optical Coherence Tomography

OCT is a light-based intravascular imaging modality that provides microscopic level visualization, which has expanded our understanding of CAV [12]. OCT offers a 10-fold greater spatial resolution than IVUS, which allows for better visualization of intimal thickening with a superior accuracy [16]. In a study by Hou et al., OCT was more sensitive than IVUS for detecting intimal hyperplasia, especially when it was less than 150 μm [26]. Another study by Garrido et al. found that OCT measurements of MIT had good correlation with IVUS measurements, with a mean difference in MIT of 0.0033 mm (95% confidence interval −0.049 to 0.043), taking advantage of lower interobserver variability (r = 0.94 for OCT vs. r = 0.78 for IVUS) and better plaque characterization [27]. Despite its many strengths, OCT requires additional contrast administration, thus increasing the risk of contrast-related complications [16,27]. More importantly, unlike IVUS, OCT has not been associated with clinical outcomes in CAV. Therefore, IVUS is a standard of care in early surveillance for CAV, and more studies are needed to identify if OCT measurements or plaque features correlate with worse outcomes in CAV patients before OCT can be endorsed by guidelines for CAV screening [24,28].

## 3. Non-Invasive Modalities

### 3.1. Anatomical Imaging

#### 3.1.1. Computed Tomography Coronary Angiography/Coronary Artery Calcium Scoring

Computed tomography coronary angiography (CTCA) is a very promising technique that the ISHLT gave a class 2b recommendation for the diagnosis of CAV [29]. The ISHLT recommends using CTCA annually or biannually as a non-invasive alternative to coronary angiography for detecting CAV in ≥2 mm epicardial vessels [30]. CTCA can accurately and safely characterize coronary vascular lesions without the risks associated with an invasive procedure, aside from contrast exposure [31]. In a study of 52 HTx patients who underwent 64-Multidetector CT with retrospective electrocardiogram (ECG) gating, before being evaluated using ICA and IVUS, CTCA had a sensitivity for CAV detection similar to that of ICA. CTCA also had a higher negative predictive value for excluding significant stenoses requiring percutaneous intervention compared to ICA (63% vs. 59%) [32]. A meta-analysis of 13 studies that used current CTCA technologies found a diagnostic accuracy of 94% for CAV, concluding CTCA to be a reliable non-invasive alternative to ICA [33].

The coronary artery calcium (CAC) score using CT is a well-established marker of coronary atherosclerosis, with a negative CAC score correlating with good clinical outcomes [34]. CAC estimates atherosclerotic plaque burden by assessing the calcified portion of coronary plaque; however, it may not detect soft plaque or intimal thickening without calcification. Given the complex pathophysiology of CAV, it is unclear whether CAC score can predict CAV and long-term outcomes [34,35]. It was previously believed that CAC offered no prognostic value because calcification was absent even in severe disease and earlier studies failed to demonstrate any predictive value for CAC in CAV patients [36,37,38]. However, a study in 2018 of 133 patients found that the absence of CAC on CT had a negative predictive value of 97% for severe forms of CAV (CAV_2–3_) and 88% for significant stenoses [37]. This study also reported a significant association between the presence of CAC and clinical outcomes at 7.5 ± 2.6 years including death, graft loss, and major adverse cardiac events.

Limitations of CTCA include radiation dosage, as HTx patients need regular periodic surveillance; however, all the methods of surveillance include radiation exposure, except for stress echocardiography [39]. Another drawback is the need for contrast, which may be of particular concern to HTx patients with pre-existing cardiorenal syndrome or renal impairment, secondary to the nephrotoxic effects of immunosuppressive medications [8]. Furthermore, higher heart rates in HTx patients may challenge image quality; however, recent advances in CT technologies helped to overcome this limitation [40,41,42].

#### 3.1.2. CT Perfusion Imaging

Although primarily an anatomical imaging modality, CT can also serve as a functional tool utilizing myocardial perfusion imaging (MPI). Dynamic CT MPI assesses the perfusion of blood to the myocardium by the intravenous administration of a contrast agent [43]. Serial CT images are taken to facilitate the measurement of myocardial attenuation over time, following pharmacologically induced stress with a vasodilator, which are then reflected as a myocardial blood flow (MBF) on volumetric perfusion maps [44].

A meta-analysis in 2018 by Lu et al. reported a pooled sensitivity of 93% (95% CI 82–98%) and specificity of 82% (95% CI 70–91%) for dynamic CT MPI in the detection of hemodynamically significant coronary artery disease [45]. However, there is a paucity of data assessing its use in the evaluation of CAV. Ahn et al. evaluated global and minimum MBF on CT MPI in 63 HTx recipients, of whom 35 (55.6%) had a diagnosis of CAV on invasive investigation [46]. Of the patients with CAV, the median MBF was significantly reduced for both global and minimum MBF (*p* < 0.01). The overall sensitivity and specificity of minimum MBF was 74% and 46%, respectively.

In addition to the limited clinical evidence for CT MPI for CAV, there are other practical limitations for this imaging modality. Artifacts, including cardiac motion artifact from tachycardia, may present as myocardial perfusion deficits and lead to a false positive result [43]. Furthermore, it utilizes higher doses of intravenous contrast, presenting a challenge in particular for those with allergies or renal insufficiency. It also requires technical expertise, a much longer scanning time than CTCA, and is not routinely available in many centers.

### 3.2. Functional Imaging

#### 3.2.1. Echocardiography

Traditionally, echocardiography has formed the cornerstone of functional cardiac evaluation. For HTx patients, rest two-dimensional and Doppler echocardiography may provide valuable insights into allograft morphological and functional alterations; however, its role in CAV diagnosis may be limited [47,48]. Significantly, baseline LVEF is often reported to be elevated in cardiac allografts, likely owing to parasympathetic denervation and an increased level of serum catecholamines. As such, normal LV systolic function may not exclude significant pathology, including CAV [49].

Alternatively, dobutamine stress echocardiography (DSE) offers a functional assessment of cardiac allografts and, as such, has been validated for the surveillance of CAV, particularly in patients with impaired kidney function who would be unsuitable for frequent angiography [50,51]. Furthermore, DSE is utilized for long-term follow-up in low-risk patients who have passed five years post-HTx without significant findings on yearly ICA.

The diagnostic power of DSE for CAV has been well studied. Spes et al. reported the diagnostic sensitivity of DSE to be 85%, with a specificity of 88% in a study of 109 HTx patients, compared with ICA and IVUS [52]. This study also reported a potential prognostic value of DSE, where an increase in the number of segments with detectable wall motion abnormalities was associated with an increased risk of cardiac events (relative risk 7.26, *p* = 0.0014) [52]. In contrast to this, a recent meta-analysis reported a pooled sensitivity of 60.2% and a specificity of 85.7% for the detection of CAV across 11 studies [53]. There was significant variability in the sample sizes of these studies, which may have contributed to the discordant findings. Furthermore, a large study of 497 HTx patients reported a negative predictive value of only 41% for any degree of CAV [54]. As such, interpretation of a negative DSE should be analyzed with caution.

Several techniques have been suggested in conjunction with DSE, to improve its diagnostic value. Regional myocardial deformation analysis (strain assessment) has been shown to increase the sensitivity of CAV detection, even in patients with negative ICA. Eroglu et al. reported an increased sensitivity of DSE from 63% to 88% in 42 HTx patients using regional myocardial deformation analysis [55]. Similar findings have been reported utilizing speckle tracking, tissue Doppler imaging, and global longitudinal strain for quantitative segmental myocardial deformation, with an improved sensitivity and negative predictive value (up to 90% and 96%, respectively) [1,56].

Limitations of DSE include variable sensitivity and negative predictive values for the detection of mild CAV, limiting it as an effective rule-out test [57]. DSE may not show ischemia in individuals with early CAV, non-flow limiting lesions, and, as such, is a missed opportunity for early intervention. These features render echocardiography less reliable for the diagnosis of CAV.

#### 3.2.2. Cardiac Magnetic Resonance

**Figure 3:**
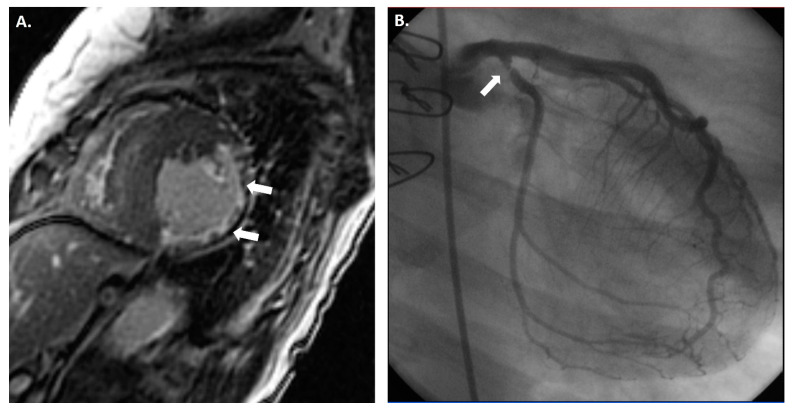
(**A**) Cardiac magnetic resonance imaging (CMR) showing clear thinning (white arrows) of the anterolateral, inferolateral, and inferior mid-cavity, with subendocardial delayed enhancement in these regions suggesting a vascular etiology. The hypertrophy of the septum is profound but is presumably compensatory due to the extensive loss of lateral wall myocardium. While this CMR finding could be multifactorial, invasive coronary angiography showed a severe obstruction (white arrow) in the proximal circumflex artery (**B**).

Cardiac magnetic resonance imaging (CMR) is a non-invasive imaging modality that can provide comprehensive information about the structure and function of the heart that is relevant for the diagnosis and prognosis of CAV (Figure 3) [8]. CMR can provide information about structural changes by measuring parameters such as extracellular volume (ECV) fraction, fibrosis, and edema [58]. However, functional parameters such as myocardial perfusion reserve (MPR), diastolic strain (DS) rate by CMR, and peak filling rate (PFR) carry more importance for CAV diagnosis, as most studies using CMR have focused on MPI and strain imaging [59,60]. Therefore, a multiparametric CMR can safely measure various parameters to detect and grade CAV by assessing the myocardial tissue and function in HTx recipients without exposing them to radiation or iodinated contrast agents [61].

#### 3.2.3. Tissue Characterization Parameters

Increased ECV reflects increased interstitial fibrosis and edema in the myocardium and, together, these parameters are associated with worse outcomes in CAV patients [62]. ECV can be measured by using T1 mapping techniques, both before and after the administration of a contrast agent. According to Sciaccaluga et al., ECV was shown to correlate to vascular stenosis on ICA and to fibrosis at endomyocardial biopsy analysis [1]. However, further studies are needed to assess whether ECV is an early sign of CAV-induced fibrosis.

T2-weighted images are sensitive for the presence of water and show edema in the myocardium. T2 mapping images are quantitative maps of the T2 values of the myocardium and can measure the extent and severity of edema. In a prospective single-center study of 99 HTx patients, Chaikriangkrai et al. showed that T2-weighted sequences can predict clinical outcomes at multivariate analysis, whilst extracellular volume and pre-contrast T1-weighted sequences tend to remain stable [62]. In their study, a higher T2 (T2 ≥ 50.2 ms) independently predicted adverse clinical outcomes (HR: 3.01; 95% CI: 1.39 to 6.54; *p* = 0.005) after adjusting for LVEF, left ventricular end-systolic volume (LVESV), and late gadolinium enhancement (LGE). Lee et al. found that CAV grade 2/3 was associated with higher T2 values and a lower LVEF than CAV grade 1, indicating more myocardial disease in the former group [61].

LGE imaging evaluates for the presence of fibrosis or edema in the myocardium by assessing the differential washout of the contrast agent from the normal and the fibrotic myocardium [63]. Fibrotic or edematous myocardium retains the contrast agent longer than normal myocardium and appear as bright regions on delayed imaging. The pattern of LGE is important, it can be focal or diffuse, and mesocardial (mid-wall) involvement is more typical in ischemic myocardium or in those with CAV. LGE has been proven to be a prognosticator in HTx patients [64]. Specifically, LGE detection of myocardial scarring has been seen to correlate with CAV, even in the absence of angiographically significant stenosis, suggesting the possibility of early diagnosis, prior to ICA detectable disease [63]. Clinically, however, focal scarring may also represent peri-procedural damage or early episodes of acute graft rejection, resulting in vascular dysfunction [63,65].

#### 3.2.4. Functional Parameters

These can be classified into systolic parameters (LVEF, stroke volume, and cardiac output) and diastolic parameters (PFR, DS, and MPR). CMR can serve as an alternative to echocardiography to assess systolic dysfunction in patients with insufficient acoustic windows, and the derived diastolic parameters may carry significant value for diagnosing and grading CAV [66]. In a prospective study by Erbel et al., 63 cardiac allograft recipients were followed using CMR, four weeks from their ICA, and PFR was found to be lower in patients with advanced CAV compared to those with earlier stages, suggesting that diastolic dysfunction may be a sensitive marker of CAV progression [67]. Moreover, early impairment of DS rates and MPR indices were associated with microvascular dysfunction, while systolic parameters were found to be normal either in early or late stages [67]. Therefore, diastolic dysfunction might be more sensitive than systolic dysfunction for the early detection of CAV [1].

Further assessment of microvascular disease can be attained by using stress perfusion CMR to estimate MPR, which represents the ratio of MBF during stress to MBF at rest. In a retrospective study of 20 HTx patients by Naranget al., an MPR ≤ 1.68 had a 100% sensitivity and 100% negative predictive value in detecting CAV; however, the authors still reported a relatively low specificity of 63% [68]. Miller et al. reported on a larger cohort of 48 HTx patients, noting that multiparametric CMR outperformed ICA for the detection of moderate CAV [66]. Overall, the detection of CAV using CMR had a sensitivity range from 41 to 100% and a specificity range from 61 to 100%, according to the pool data from eight studies in the systematic review by Ajluni et al. [69].

CMR also allows for the qualitative and semi-quantitative assessment of myocardial blood flow, which may assist in the detection and surveillance of CAV [70]. Prior investigations have shown the MPR index on CMR to have a diagnostic value for both epicardial and microvascular CAV, compared to an invasive assessment [66]. This has been validated in multiple studies, both prospective and retrospective, in which MPR was demonstrated to have a diagnostic sensitivity of 100% and a specificity of 80–87% [59,60]. Conversely, the qualitative assessment of myocardial perfusion appears to have only modest sensitivity and specificity (41% and 74%, respectively) in CAV detection [71]. Furthermore, significant expertise is required for the interpretation of MPI with CMR and this specific knowledge is often not easily accessible for routine CAV surveillance. [72].

While excellent for tissue characterization and early changes in CAV, CMR does have some limitations. The first is access and cost and, additionally, those with significant claustrophobia may not tolerate the prolonged scanning time. Patients with more advanced kidney impairment would not be suitable for gadolinium enhancement, with risk for nephrogenic systemic sclerosis [73]. Finally, the presence of metal prostheses may contraindicate CMR and the presence of cardiac pacemakers may introduce artifacts and make the images more challenging to interpret.

### 3.3. Radionuclide Myocardial Perfusion Imaging

#### 3.3.1. Single-Photon Emission Computed Tomography

Single-photon emission computed tomography (SPECT) imaging is a form of MPI which creates a three-dimensional representation of the myocardium based off sequential planar slices [74]. It utilizes the intravenous administration of a radioactive isotope, which emits gamma photons [75]. Following exercise or pharmacologically induced coronary artery dilation, SPECT imaging is used to identify areas of myocardium with hemodynamically significant perfusion deficits, visualized as a reduced radiotracer concentration [76]. When conducted with hybrid SPECT/CT machines, the concomitant assessment of LV volume and function may assist enhancing the diagnostic accuracy [77]. Studies have shown the incremental value of stress SPECT imaging over baseline rest SPECT imaging for the detection of CAV and potential prognostication [78,79].

The diagnostic power of SPECT imaging for CAV is variable and appears to be dependent on both the class of the radiotracer used and the method of cardiac stress [11,80,81]. Previously, Ciliberto et al. reported a sensitivity of 67% and a specificity of 100% for the diagnosis of CAV in 50 HTx patients, utilizing exercise thallium scintigraphy [82]. Rodney et al. found no significant difference in the sensitivity of Thallium-201 compared to technetium 99m in the detection of CAV in 25 HTx patients following exercise [83].

SPECT following pharmacological stress has also been investigated for this population. Carlsen et al. utilized dipyridamole to induce stress in 67 post-transplant patients, finding the negative predictive value of a normal 99m-technicium sestamibi/tetrofosmin SPECT scan to be 98% [81]. Wu et al. have subsequently reported a sensitivity of 89% and a specificity of 71% for the detection of significant CAV with thallium-201 SPECT imaging in 50 heart transplant recipients following dobutamine load [84]. Furthermore, their study found a prognostic association, reporting that patients with a large reversible perfusion defect on SPECT had a significantly higher risk of cardiac death at 40 months post-transplant (*p* = 0.002). Elhendy et al. also demonstrated significant prognostic value of stress SPECT MPI on 166 heart transplant patients. Overall, they presented a significant relationship between abnormalities of myocardial perfusion and cardiac death at three years post-transplant (relative risk ratio of 2.5, 95% CI 1.6–11.7, *p* = 0.002) [85]. This same cohort was observed for long-term outcomes, with abnormal SPECT MPI holding significant prognostic implications up to five years post-transplant for all-cause mortality (HR 1.61, 95% CI 1.01–2.56, *p* = 0.037) [86].

In addition to this semi-quantitative visual assessment, Aguila et al. presented the diagnostic and prognostic value of a quantitative perfusion assessment using total perfusion deficit (TPD) on stress SPECT [79]. In a large retrospective study of 503 HTx patients, they reported TPD to have equivocal diagnostic accuracy for CAV to traditional semi-quantitative SPECT imaging. Significantly, however, they noted stress TPD to be the only independent predictor of mortality at five years post-HTx (adjusted HR, 1.07; *p* = 0.030). Perfusion quantification with TPD has also been found to have a lower inter-observer variability than semi-quantitative visual assessment, making it more reliable for CAV surveillance [79,87].

SPECT imaging has some limitations, including radiation exposure and the potential to under-estimate obstructive epicardial coronary disease [75,88,89,90]. Logistically, perfusion defects are assessed compared to the greatest area of radiotracer uptake in each resting or stress scan, meaning all quantification is relative. As such, patients with diffuse disease may display “balanced ischemia”, which may be reflected in a normal-appearing scan [89].

#### 3.3.2. Positron Emission Tomography

**Figure 4:**
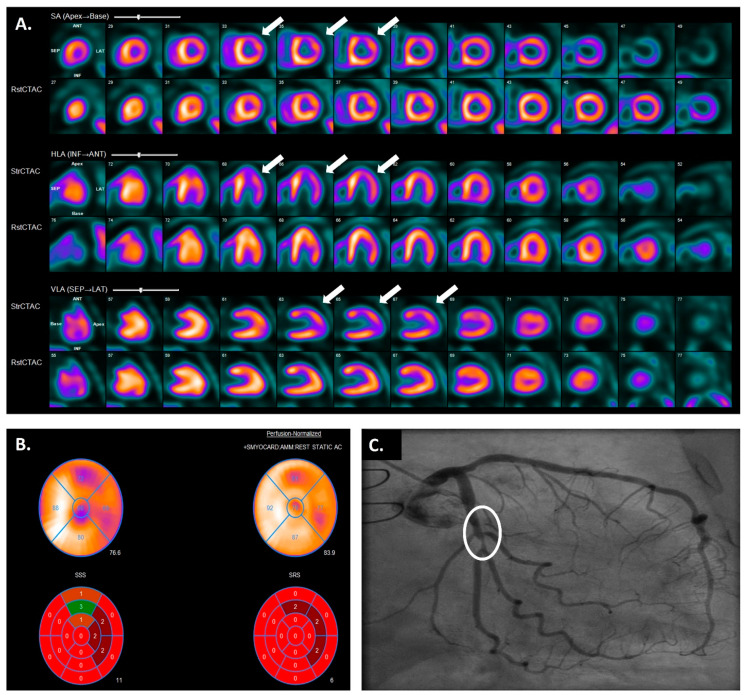
Moderate to large reversible perfusion defect (white arrows) in the anterior and inferolateral wall showed using positron emission tomography (**A**) and computed tomography cardiac perfusion test (**B**). Coronary allograft vasculopathy was confirmed with invasive coronary angiography; this study showed a severe obstruction (white circle) in the mid circumflex artery (**C**).

Though less widely available than SPECT, positron emission tomography (PET) is considered the gold-standard modality MPI, with the greatest accuracy, Figure 4 [91]. PET also utilizes the injection of radio-isotope-labeled drugs; however, these act by emitting positrons in target tissue [92]. PET allows for both the qualitative and quantitative assessment of myocardial perfusion [72]. Typically, contemporaneous CT imaging provides anatomical mapping [93]. Different radio-isotopes are used compared to SPECT, usually rubidium-82 or ammonia-13, to assess perfusion and, due to their short half-lives, usually require an onsite generator or cyclotron [93].

PET imaging has a superior temporal and spatial resolution compared to SPECT imaging, resulting in a more accurate attenuation correction. It allows for the racking of dynamic tracer activity through time, permitting absolute quantification of perfusion; MBF [93]. In stress studies, the ratio of MBF at maximal stress to that at rest is referred to as the myocardial flow reserve (MFR) [72]. HTx patients have a higher resting MBF than the baseline population on PET quantification [94].

Compared to SPECT imaging, which may underestimate the degree of CAV due to homogenous “balanced ischemia”, PET can more accurately detect and quantify diffuse perfusion deficits [80]. Multiple studies have established the diagnostic accuracy of PET for CAV evaluation, compared to invasive coronary angiography, with an overall sensitivity of 69–97% and a specificity of 56–97% [95,96,97]. Furthermore, studies have suggested that dynamic PET may have utility in detecting microvascular changes that precede the clinical and angiographic detection of CAV [98].

There is growing evidence supporting the prognostic significance of PET in patients with CAV [95]. Mc Ardle et al. found that patients with abnormal Rubidium-82 PET uptake after dipyridamole stress (reflected as MFR ≤ 1.75) had four times higher risk of adverse cardiac events (HR 4.41, 95% CI 1.53–12.73, *p* = 0.006) and mortality (HR 6.4, 95% CI, 1.62–25.7, *p* = 0.008) [99]. More recently, Wiefels et al. found a significant relationship between impaired MBF on stress PET within the first three years of transplant and all-cause mortality (*p* = 0.03) [100]. This study further demonstrated an incremental association with persistently impaired MBF on repeat PET scan (within six years of transplant) and reduced event-free survival; however, this result was not statistically significant (HR 2.06, 95% CI 0.96–4.45, *p* = 0.067). Interestingly, early impairment of MBF on PET, within the first few months of transplant, does not appear to be associated with early CAV and is often seen to normalize within one year [80,101].

Compared to SPECT imaging modalities, PET is more accurate and has a lower radiation burden (usually less than 5mSv, depending on body weight) [76,93]. It is also the most accurate MPI modality in obese patients. Another benefit of PET imaging is the short acquisition time and short half-life of radio-isotopes, usually allowing for rest and stress imaging to occur in the same session [72,102]. One of the main drawbacks of PET is cost, with equipment and radiotracers typically being more expensive than those required for SPECT [102].

## 4. Biomarkers

Recent studies have focused on the identification of biomarkers that may help predict and risk stratify HTx patients for CAV. Considering that both immunological and non-immunological inflammatory processes may be involved in the pathogenesis of CAV, a wide range of potential biomarkers, both of the HTx recipient and the donor, may be potential targets for evaluation [103].

Many studies have evaluated traditional markers of coronary artery disease in HTx patients with CAV. Elevated total cholesterol, low-density lipoprotein, and triglyceride levels have all been associated with severe intimal thickening in HTx recipients, consistent with CAV [104,105,106]. Elevated brain-natriuretic peptide (BNP) levels after transplant have also been found to independently predict the development of CAV [107]. Furthermore, Szyguła-Jurkiewicz et al. retrospectively reviewed 198 HTx patients, reporting an independent association between pre-transplant N-terminal pro-BNP and the risk of developing CAV (OR 16.46, *p* < 0.001) C-reactive protein (CRP) may also be associated with CAV; however, it is unclear if it is predictive or specific to this condition [104,108].

Recent studies have evaluated the clinical and prognostic utility of high-sensitivity troponin I (hs-Tni), which has well documented value in non-transplant cardiovascular outcomes [109,110]. Patel et al. recently reported hs-Tni levels in 156 HTx patients, noting a significant and incremental association with serum hs-Tni levels and the severity of CAV (*p* = 0.016 on multivariate analysis) [111]. Furthermore, patients with elevated hs-Tni > 4.9 pg/mL had a higher risk of adverse events ten years post-transplant, including death or re-transplantation, even after adjustment for age, body mass index, systolic graft function, renal function, and diabetes (HR 1.80; 95% CI 1.15–2.83, *p* = 0.01) [111]. Furthermore, lipoprotein-a (Lp(a)) has been evaluated in HTx patients, with Enriquez-Vazquez et al. reporting elevated Lp(a) ≥ 50 mg/dL to be associated with CAV at one year post-transplant [112]. Given the high availability of hs-Tni and Lp(a) measurements, these may prove to be useful biomarkers for CAV, with further prospective evaluation.

A study in 2020 by Bjerre et al. further evaluated 91 cardiovascular disease related proteins, identifying two novel, potentially significant recipient biomarkers [113]. Specifically, HTx patients with diagnosed CAV had significantly deranged serum levels of subtilisin/kexin type 9 (PCSK9) and paraoxonase 3 (PON3), both of which are involved in cholesterol homeostasis [113]. This adds evidence to the hypothesis that, despite its differences with native heart atherosclerosis, cholesterol accumulation is significant in the pathogenesis of CAV. This study also reported significantly elevated N-terminal pro-BNP (NT-proBNP) in patients with microvascular CAV; however, there was no association with clinical outcomes in these patients.

Furthermore, an initial study by Almufleh et al. identified 14 potential novel biomarkers for the detection of CAV [114]. This group then further evaluated the diagnostic power of three specific proteins, intercellular adhesion molecule 2 (ICAM), receptor tyrosine-protein kinase ErbB3 (ERB), and tissue factor (TF). In patients with confirmed CAV on IVUS, abnormal ICAM and ERB had a combined diagnostic sensitivity of 47% and a specificity of 90% [115].

Similar to other solid organ transplantations, cytomegalovirus (CMV) infection is associated with increased morbidity and mortality. Specifically, CMV infection (both symptomatic and asymptomatic) post-transplant has been found to significantly increase the risk of CAV and subsequent CAV-related death and graft loss [116,117]. Furthermore, Hussain et al. presented a direct relationship between pre-transplant CMV status and the long-term risk of CAV, all-cause mortality, and cardiac mortality in 165 pediatric HTx patients over a follow-up of more than 10 years [118].

Regarding donor biomarkers, both prospective and retrospective studies have evaluated the potential utility of donor-derived, cell-free DNA in predicting and prognosticating CAV. Overall, currently no clinical association has been identified [119]. Ongoing research is aimed at identifying other potential biomarkers including the presence of specific micro-RNAs, serum interleukin levels, and tumor necrosis factors. However, despite substantial progress, there are currently no validated biomarkers for detection or prognostication in CAV [9].

## 5. Future Directions

The role of artificial intelligence (AI) in the early detection of CAV is not yet established; however, it is an area of ongoing research and innovation [120]. In a review article about the current state of artificial intelligence in cardiac transplantation, Goswami suggests that AI could be used to analyze image segmentation of cardiac biopsies, as well as genomic and proteomic data, to identify new factors that influence the development and progression of CAV [121]. Applying machine learning and deep learning algorithms to various non-invasive imaging modalities can analyze large and complex datasets, extract relevant features, and learn patterns that are associated with CAV. For example, AI-enhanced echocardiography has the potential to automatically and accurately measure GLS and other strain parameters, which are sensitive indicators of subclinical myocardial dysfunction and predictors of CAV development and outcomes [34]. Moreover, AI algorithms can analyze the patterns of strain curves throughout the cardiac cycle and classify them into physiological, non-physiological, or uncertain categories [34,122,123], which may help to differentiate CAV from other causes of cardiac dysfunction.

In the meta-analysis by Alskaf et al., deep learning has shown promise to improve CMR MPI diagnostic accuracy, which can be used as an indicator for CAV progression [124]. The deep learning-based automated quantification of CAC, a predictor of CAV severity, efficiently extracted CACs from CTCA and reliably assigned categorical classification for Agatston scores, without additional radiation exposure [125]. In addition, the deep learning system studied in a multicenter study by Lin et al. provided rapid measurements of plaque volume and stenosis severity from CTCA that agreed closely with expert readers and IVUS [126]. The prototype deep learning system studied by Su TY et al. for myocardial ischemia auxiliary diagnosis using SPECT MPI showed a considerably reduced time required for image interpretation which can help provide an accurate and timely diagnosis of CAV [127]. Given these results, AI has the potential to provide useful information on the severity and prognosis of CAV, as well as the differentiation of CAV from other cardiac conditions. More research is ongoing to further establish its potential in enhancing the early detection and surveillance of CAV in the clinical setting.

## 6. Discussion and Clinical Implications

CAV remains a major challenge for the long term-success of heart transplants. Early diagnosis and prevention are of paramount importance. Several imaging modalities exist for CAV screening, each with advantages and limitations (Table 2). There may be some variation for non-invasive imaging modality choice, depending on several factors, including the patient’s risk profile, degree of renal dysfunction, local availability and expertise, and cost.

**Figure 5:**
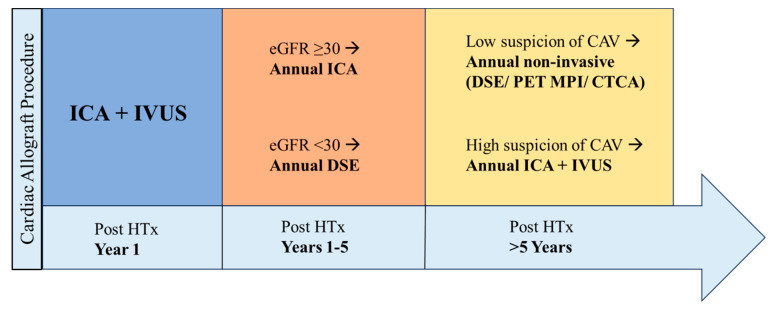
**CAV surveillance over time based on expert opinion**. *CAV, cardiac allograft vasculopathy; HTx, heart transplant; ICA, invasive coronary angiography; IVUS, intravascular ultrasound; DSE, dobutamine stress echocardiography; PET, positron emission tomography; MPI, myocardial perfusion imaging; CTCA, computed tomography coronary angiography*.

A common clinical approach based on expert opinions for CAV surveillance (Figure 5) involves performing an ICA + IVUS at the one-year mark post-transplant. A positive test on either modality (CAV_1/2_ on ICA or MIT > 0.5 µm on IVUS) may warrant alteration of medical management or intervention, in an attempt to slow the progression of CAV. This may include escalating immunosuppressive treatment to mammalian target of rapamycin (mTOR) inhibitors, in conjunction with statins or intervention through percutaneous coronary intervention or re-transplantation, depending on the severity of CAV [128,129]. A negative test, however, will defer the use of IVUS with the continuation of an annual ICA until the five-year mark, provided preserved kidney function (eGFR ≥ 30 to 40 mL/min/1.73 m^2^). For HTx recipients with significant kidney disease (e.g., eGFR < 30 to 40 mL/min/1.73 m^2^), an annual DSE can be an alternative within the first five years.

Annually, once the recipient has passed the five-year mark post-transplant, a non-invasive modality is usually used. While annual DSE has been frequently used in the past with its favorable cost–benefit ratio, accessibility, and lack of radiation, PET MPI or CTCA are being increasingly used for annual long-term surveillance after the five-year mark. Nonetheless, for patients with a higher clinical index of suspicion or evidence of CAV on ICA, annual surveillance with ICA ± IVUS should be continued, if renal function permits.

## 7. Conclusions

Ongoing advances in non-invasive imaging hold promise for the improved early detection and diagnostic accuracy of CAV; however, current guidelines still recommend ICA ± IVUS as the gold standard, particularly in the first five years post-heart transplant. Although many non-invasive imaging modalities have been studied for this purpose, there is currently no consensus guideline for first line investigation, although PET is favorable for MPI due to its accuracy and CTCA holds promise as a non-invasive anatomic imaging modality in appropriately selected patients. The rapidly evolving era of AI and the development of new biomarkers may revolutionize the way we approach the diagnosis and surveillance of CAV.

## Figures and Tables

**Table 1:** International Society for Heart and Lung Transplantation (ISHLT) classification for cardiac allograft vasculopathy grading.

ISHLT CAV Grade	ICA Findings
**CAV_0_ (not significant)**	Undetectable stenosis/lesion
**CAV_1_ (mild)**	Left main stenosis < 50%, *and/or:*
	Primary vessel lesion < 70%, *and/or:*
	Secondary or isolated branch stenosis < 70%
**CAV_2_ (moderate)**	Left main stenosis < 50%, *and/or:*
	Primary vessel lesion > 70%, *and/or:*
	Isolated branch stenosis in two vascular territories > 70%
**CAV_3_ (severe)**	Left main stenosis ≥ 50%, *and/or:*
	At least two primary vessel lesions ≥ 70%, *and/or:*
	Branch stenoses in all three vascular territories ≥ 70%, *and/or:*
	CAV_1_ or CAV_2_ with allograft dysfunction (LVEF ≤ 45%)

Adapted from the 2010 International Society for Heart and Lung Transplantation. Proximal vessel includes the proximal and middle third of LAD, left circumflex, dominant, or co-dominant RCA. Secondary branches include the distal third of the LAD or left circumflex, any obtuse or marginal branches, or any portion of non-dominant RCA. *CAV, cardiac allograft vasculopathy; ICA, invasive coronary angiography; LAD, left anterior descending artery; RCA, right coronary artery; LVEF, left ventricular ejection fraction* [2,3,13].

**Table 2:** Summary of advantages and limitations of different diagnostic modalities used for CAV surveillance.

Diagnostic Modality	Advantages	Limitations
Invasive coronary angiography (ICA)	Widely available Gold standard for CAV diagnosis Can measure fractional flow reserve (FFR) to assess the functional significance of stenosis	Invasive Contrast injection Insensitive to early CAV (diffuse lesions) Cannot characterize vessel walls or lesions Requires additional procedures (IVUS, OCT) for better evaluation
Intravascular ultrasound (IVUS)	More sensitive than ICA for detecting early CAV (intimal thickening) Provides detailed vessel wall thickness measurements	Less available than ICA Invasive Increases risk of complications during angiography Lengthens procedure time and cost Limited vessel coverage
Optical coherence tomography (OCT)	Highest resolution—provides microscopic views of vessels More accurate for detecting early intimal thickening (<150 μm) Better plaque characterization compared to IVUS	Less available than ICA Invasive Requires additional contrast, increasing complication risks Not currently linked to clinical outcomes in CAV patients (unlike IVUS)
Computed tomography coronary angiography/coronary artery calcium scoring (CTCA/CAC score)	Non-invasive High diagnostic accuracy for CAV detection	Radiation exposure Requires contrast injection High heart rate can affect image quality
CT perfusion imaging	Non-invasive Functional assessment of myocardial blood flow (MBF)	High false positive rate due to motion artifacts Requires high contrast dose Time-consuming and requires technical expertise
Echocardiography/dobutamine stress echocardiography (DSE)	Non-invasive and readily available Offers functional assessment of cardiac allografts DSE may be useful for surveillance in high-risk patients (renal dysfunction) or long-term follow-up	Limited role in mild CAV diagnosis Variable sensitivity and negative predictive value for CAV detection (DSE)
Cardiac magnetic resonance (CMR)	Non-invasive and radiation-free Provides comprehensive structural and functional information Multiparametric assessment for tissue characterization (ECV, T2, LGE) and function (MPR, DS rate, PFR) Sensitivity to microvascular dysfunction Diagnostic accuracy for CAV with MPR (stress perfusion CMR)	Relatively expensive and not widely available Requires expertise for image acquisition and interpretation Variable negative predictive value for CAV detection Not suitable for patients with claustrophobia, advanced kidney impairment, or certain metallic implants
Single-photon emission computed tomography (SPECT)	Non-invasive Holds prognostic value	Radiation exposure Variable diagnostic accuracy for CAV detection Potential for false positives (obesity) “Balanced ischemia” in diffuse disease may lead to normal-appearing scans
Positron emission tomography (PET)	Non-invasive Gold-standard modality for MPI Superior spatial and temporal resolution/lower radiation burden compared to SPECT Accurate detection of diffuse perfusion deficits Holds prognostic value	Less widely available/more expensive than SPECT Requires on-site generator or cyclotron for radio-isotope production
Biomarkers	Non-invasive Holds promise for accurate non-invasive diagnosis and risk stratification of CAV	Mostly in early stages of research, requires further validation

## Data Availability

Data are contained within the article.
